# Rapid coupling between vasculature and neurons through mechanosensitive channels in the olfactory lobe

**DOI:** 10.3389/fnhum.2024.1435859

**Published:** 2024-10-07

**Authors:** Yilin Zhao, Yitong Lian, Haibo Di, Weiqiao Zhao

**Affiliations:** ^1^International Vegetative State and Consciousness Science Institute, Hangzhou Normal University, Hangzhou, China; ^2^School of Basic Medical Sciences, Hangzhou Normal University, Hangzhou, China

**Keywords:** vasculo-neuronal coupling, mechanosensitive, Piezo2, neural oscillations, olfactory

## Introduction

Neural oscillations are a prevalent phenomenon observed in various brain regions such as the cerebral cortex, hippocampus, olfactory bulb, and thalamus, playing essential roles in functions like wakefulness, sensory information processing, memory, attention, and emotion regulation (Hyafil et al., 2015; Hanslmayr et al., 2019). These oscillations, reflecting the coordinated and regulated activity of neuronal clusters, are driven by the rhythmic activity of neurons and can be categorized into different types based on their response patterns to stimuli: resting-state, evoked, induced, and entrained oscillations (Henao et al., 2020). Historically, considerable research has focused on neural oscillations induced by external stimuli such as visual, auditory, and olfactory signals (Deng et al., 2024; Xu et al., 2021). More recently, the synchronization of brain network oscillations by internal rhythmic signals has attracted increasing attention (Engelen et al., 2023). Interoception, or the perception of internal bodily signals, plays a significant role in sensory awareness and has been suggested to contribute to emotional regulation and the formation of self-consciousness (Tallon-Baudry, 2023; Hsueh et al., 2023; Feldman et al., 2024). Among the sources of interoceptive signals, the cardiovascular system has been extensively studied, revealing phenomena such as neurovascular coupling, vasculo-neural coupling, and cortical responses to heartbeats. Extensive research on neurovascular and vasculo-neural coupling has established the cardiovascular system's influence on brain activity via neurotransmitters and vasoactive substances (Silvani et al., 2016; Kim et al., 2016). However, groundbreaking findings published in February 2024 have proposed a novel interaction mechanism (Jammal Salameh et al., 2024). This study demonstrated that cardiovascular pulsations, specifically those from the cardiac cycle, directly influence the olfactory bulb's neuronal excitability through mechanosensitive ion channels, independent of traditional synaptic transmission. These findings suggest a fundamental shift in understanding how blood pressure fluctuations can modulate brain function.

## Mechanosensitivity in neural oscillations

The working heart-brainstem preparation (WHBP), developed in 1995, serves as a valuable platform for elucidating the neural mechanisms governing respiratory and cardiovascular systems and their integration in both health and disease (Paton et al., 2022). Veronica Egger's research group has created an innovative semi-intact perfused nose-olfactory bulb-brainstem preparation in rats. This model, retaining both the olfactory lobe and brainstem, is particularly advantageous for examining oscillatory activity in the olfactory lobe, independent of other cortical regions and peripheral organs. Their prior studies revealed that spontaneous slow oscillations in the olfactory bulb's field potentials could be detected even in the absence of odor molecules, respiratory rhythm interference, and inputs from olfactory processing cortices, such as the piriform cortex, as well as synaptic transmissions from limbic structures, including the hippocampus (Pérez de los Cobos Pallarés et al., 2015). To elucidate the underlying mechanisms of these phenomena, Jammal Salameh et al. (2024) conducted a series of experiments to investigate the causes. The researchers used a peristaltic pump to simulate the periodic pressure changes caused by blood flow into the brain during heartbeats. By analyzing the spectral characteristics of field potentials in the mitral cell layer and comparing them to the pulse fluctuations, they established a correlation between the main frequencies of the pulse rhythm and the field potentials. This correlation was disrupted when pressure pulsations were minimized, indicating that the field potential oscillations in the olfactory bulb were indeed caused by periodic pressure pulsations in the vessels. Further analysis using hypoxic perfusion methods, which reduce neuronal activity, led to the disappearance of local field potential (LFP) oscillations, supporting the neurophysiological origin of these signals. Statistical analysis of the harmonics of the field potential signals also suggested that the oscillatory signals were mediated directly by pressure receptors rather than synaptic transmission. The study further examined the spatial-temporal stability of these oscillation signals, finding that significant harmonic signals occurred only in the mitral cell layer (MCL) and maintained their frequency and amplitude consistently across different locations within the MCL. This robustness suggests that the pressure-sensitive field potential oscillations produced in the olfactory bulb MCL are mediated by Piezo mechanosensitive channels, especially Piezo2, which is highly suitable for tracking vascular pulse pressure waves due to its fast activation and deactivation kinetics. The contribution of Piezo2 was further confirmed by pharmacological intervention and computer simulation experiment. Finally, *in vivo* experiments corroborated the role of vascular pulse regulation of neuronal excitability in the olfactory bulb. This study, utilizing a combination of semi-intact preparations and *in vivo* recordings, highlighted that arterial blood pressure pulsations can significantly influence neuronal excitability through a direct mechanotransductive pathway, mediated by Piezo mechanosensitive ion channels.

## Discussion

**Figure 1:**
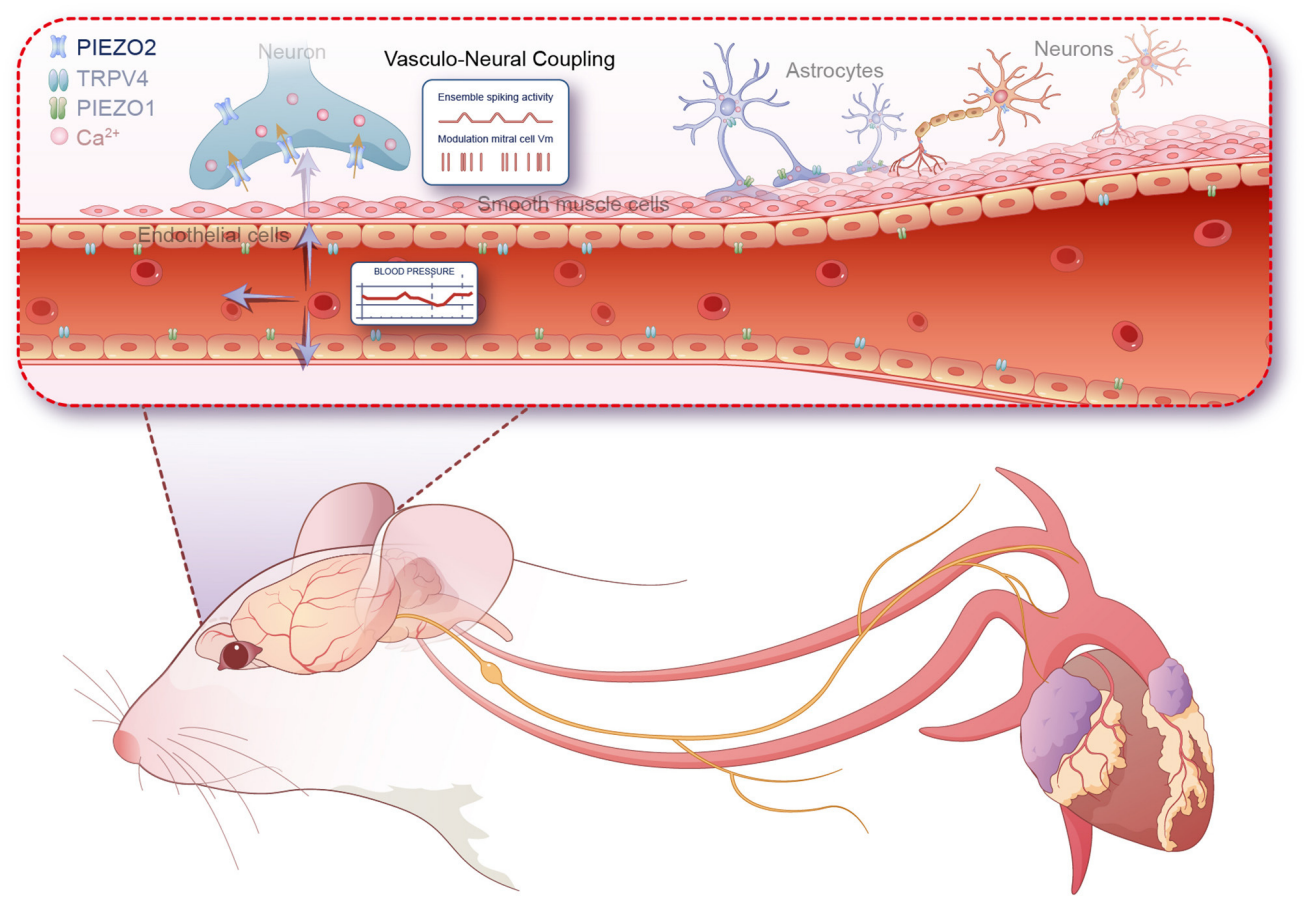
Mechanism by which perivascular neurons in the olfactory bulb rapidly respond to blood pressure pulsations via Piezo2 channels.

This finding suggests synchronized rhythmic oscillations are induced in the brain, mediated by mechanoreceptors, offering a new perspective on the interaction between the heart and brain. As a key component of the neurovascular coupling unit, endothelial cells sense shear stress or vascular pressure through a series of molecular mechanisms, thereby regulating vascular function and maintaining overall hemodynamic stability (Aitken et al., 2023). This process plays a crucial physiological role in regulating vascular tone, vascular remodeling, inflammatory responses, and endothelial cell function. During the process of sensing shear stress, endothelial cells release substances that affect neuronal activity, such as nitric oxide (NO), ATP, and prostaglandins (Bahr-Hosseini and Bikson, 2021). Whether these substances can indirectly regulate neuronal activity through astrocytes to achieve vasculo-neuronal communication is a question worth exploring. Besides endothelial cells, astrocytes also express various mechanosensitive channels that can directly sense vascular pressure (Turovsky et al., 2020). Studies have found that astrocytes respond to vascular pressure changes via TRPV4 and Piezo1 channels, leading to an increase in intracellular Ca^2+^, which in turn triggers the release of ATP, glutamate, and other substances that affect surrounding neurons (Kim et al., 2016; Chi et al., 2022). In contrast to these mechanisms, this study importantly highlights that arterial blood pressure pulsations transmitted through the cerebral vascular system can directly modulate neuronal excitability in the olfactory bulb without the involvement of other cellular components. This study importantly highlights that arterial blood pressure pulsations transmitted through the cerebral vascular system can modulate neuronal excitability in the olfactory bulb without the need for synaptic input. Mechanosensitive ion channels, particularly those belonging to the Piezo family, are instrumental in this process. These channels respond to the mechanical stretch or stress induced by blood pressure pulsations, converting these physical stimuli into electrical and biochemical signals within neurons. This discovery underscores a fast, intrinsic interoceptive mechanism that could play a critical role in how physiological processes such as blood pressure directly influence brain function and perception, particularly during states of arousal or alertness. Moreover, the study's findings offer new insights into the olfactory system's neural dynamics. The olfactory cortex's rhythmic oscillations, crucial in olfactory-related information processing such as olfactory learning, sensitivity, and associative memory in animals, are influenced by various frequency ranges including beta (15–30 Hz in rodents), gamma (40–100 Hz in rodents), and theta (4–12 Hz in rodents) oscillations. These bands are induced by respiratory rhythms, nasal inhalation flow, and olfactory molecular stimuli, highlighting the complex interplay between physiological and neural processes. In conclusion, the recent findings underscore a critical, fast-responding interoceptive mechanism through which physiological signals like blood pressure pulsations can impact neuronal activity as illustrated in Figure 1. This pathway, which operates independently of traditional synaptic interactions, invites further exploration into its role in sensory perception, emotional regulation, and potentially the formation of self-awareness. The implications of these interactions extend beyond the olfactory system, suggesting that similar mechanisms could be active in other sensory and cognitive contexts, which could be a fruitful area for future research. However, this study presents several issues that warrant further attention. Firstly, while the authors propose Piezo2 as the candidate mechanosensitive channel mediating pressure pulsations, the use of non-specific blockers in pharmacological intervention experiments introduces some uncertainty regarding this conclusion. Secondly, *in vivo* experiment results indicate that only 15% of the recorded spikes are synchronized with the heartbeat, a proportion significantly lower than those synchronized with breathing. This discrepancy prompts inquiries into whether such signals are too subtle and are obscured by background noise, or if they might integrate into other networks in a different manner. Determining whether these slow oscillatory local field potentials (LFP) represent merely an intriguing epiphenomenon or play a more substantial role remains a challenge. Utilizing conditionally knocked-out Piezo2 mice may provide more definitive answers to these questions. The findings from this study also provide significant insights into the mechanisms of interoception. Interoception is foundational to the subjective experience of one's body, which is closely linked to self-consciousness (Tallon-Baudry et al., 2018). The fact that these changes in the olfactory bulb do not require synaptic input and are directly modulated by mechanical forces provides a new understanding of how the body communicates internally. This could imply that our bodily states directly contribute to shaping our moment-to-moment perception of self, thereby influencing self-awareness in significant ways. These insights open up new areas of exploration into how other sensory systems might be similarly influenced by physiological processes and how these interactions might affect the continuous process of self-consciousness development (Park and Blanke, 2019). This could lead to a deeper understanding of the relationship between physiological states and mental states, potentially informing therapeutic strategies for disorders where these processes are disrupted.
